# The WISDOM Radar: Unveiling the Subsurface Beneath the ExoMars Rover and Identifying the Best Locations for Drilling

**DOI:** 10.1089/ast.2016.1532

**Published:** 2017-07-01

**Authors:** Valérie Ciarletti, Stephen Clifford, Dirk Plettemeier, Alice Le Gall, Yann Hervé, Sophie Dorizon, Cathy Quantin-Nataf, Wolf-Stefan Benedix, Susanne Schwenzer, Elena Pettinelli, Essam Heggy, Alain Herique, Jean-Jacques Berthelier, Wlodek Kofman, Jorge L. Vago, Svein-Erik Hamran

**Affiliations:** ^1^ LATMOS/IPSL, UVSQ Université Paris-Saclay, UPMC, Paris 06, CNRS, Guyancourt, France.; ^2^ Lunar and Planetary Institute, Houston, Texas.; ^3^ Technische Universitat Dresden, Dresden, Germany.; ^4^ Laboratoire de Géologie de Lyon, Université Claude Bernard Lyon 1/CNRS/ENS Lyon, Villeurbanne, France.; ^5^ Open University Centre for Earth Planetary Space and Astronomical Research, Milton Keynes, Milton Keynes, United Kingdom.; ^6^ Universita degli Studi Roma Tre Dipartimento di Matematica e Fisica, Roma, Italy.; ^7^ University of Southern California Viterbi School of Engineering, Los Angeles, California.; ^8^ Université Grenoble Alpes, IPAG, F-38000 Grenoble; CNRS, IPAG, F-38000, Grenoble, France.; ^9^ European Space Agency, ESA/ESTEC (HME-ME), Noordwijk, The Netherlands.; ^10^ Forsvarets forskningsinstitutt, Kjeller, Norway.; ^11^ Space Research Centre, PAN, Warsaw, Poland.

## Abstract

The search for evidence of past or present life on Mars is the principal objective of the 2020 ESA-Roscosmos ExoMars Rover mission. If such evidence is to be found anywhere, it will most likely be in the subsurface, where organic molecules are shielded from the destructive effects of ionizing radiation and atmospheric oxidants. For this reason, the ExoMars Rover mission has been optimized to investigate the subsurface to identify, understand, and sample those locations where conditions for the preservation of evidence of past life are most likely to be found. The Water Ice Subsurface Deposit Observation on Mars (WISDOM) ground-penetrating radar has been designed to provide information about the nature of the shallow subsurface over depth ranging from 3 to 10 m (with a vertical resolution of up to 3 cm), depending on the dielectric properties of the regolith. This depth range is critical to understanding the geologic evolution stratigraphy and distribution and state of subsurface H_2_O, which provide important clues in the search for life and the identification of optimal drilling sites for investigation and sampling by the Rover's 2-m drill. WISDOM will help ensure the safety and success of drilling operations by identification of potential hazards that might interfere with retrieval of subsurface samples. Key Words: Ground penetrating radar—Martian shallow subsurface—ExoMars. Astrobiology 17, 565–584.

## 1. Introduction

The search for evidence of life on Mars is the principal objective of the 2020 European Space Agency (ESA, 2013)–Roscosmos ExoMars Rover mission. If such evidence is to be found, it will most likely be in the subsurface, where organic molecules are shielded from the destructive effects of ionizing radiation and atmospheric oxidants. Despite the enormous volume of data acquired by previous orbital and landed missions, the nature and structure of the shallow subsurface (<5 m) remain largely unknown. Until now, our insights have been limited to the stratigraphy exposed in outcrops and crater walls and the composition of samples acquired within ∼1 to 10 cm of the surface (*e.g.,* Grotzinger *et al*., 2005, 2015; Vaniman *et al*., 2014; Freissinet *et al*., 2015). For this reason, the ExoMars Rover mission has been optimized to investigate the subsurface to identify, understand, and sample those locations where evidence of past life is most likely to be found.

To fulfill its objectives, the ExoMars Rover will be equipped with a variety of instruments dedicated to the study and characterization of the planet's surface and shallow subsurface (Vago *et al*., 2017). These investigations will help identify the best locations for collecting samples with the rover's 2-m drill, which will then be analyzed by a suite of onboard analytical instruments. The most fundamental requirement for the geologic characterization of the ExoMars landing site will be to understand its nature and structure, which will provide invaluable insights into its origin and evolution.

The Water Ice Subsurface Deposit Observation on Mars (WISDOM) radar has been selected as part of the ExoMars Rover mission Pasteur payload (Vago *et al*., 2017). It has been designed to provide information about the nature of the shallow subsurface over a depth range (∼3–10 m, depending on the dielectric properties of the regolith) that is both accessible and relevant to the search for traces of life. It will address some of the most important science questions about the nature of the landing site, such as its depositional and erosional history, deformational and structural development, and the potential role (and distribution) of liquid water and ice in the evolution of the local landscape. In combination with Pasteur payload's other instruments that will be used in the area survey, WISDOM will help understand the three-dimensional (3D) geological context so that the science team can assess the scientific interest of locations investigated by the rover and choose the best site and depth at which to collect samples. In addition, WISDOM will detect potential buried hazards that might jeopardize drilling activities.

This article presents the WISDOM scientific objectives, a description of the instrument, and its expected performances in relevant environments. A section is also dedicated to the measurement scenario and the data products that will be delivered during the mission. Last, examples of both simulated data and experimental data, acquired by a prototype that is representative of the WISDOM flight model, are shown to illustrate how WISDOM can contribute to the success of the mission.

### 1.1. Team organization

**FIG. 1.**
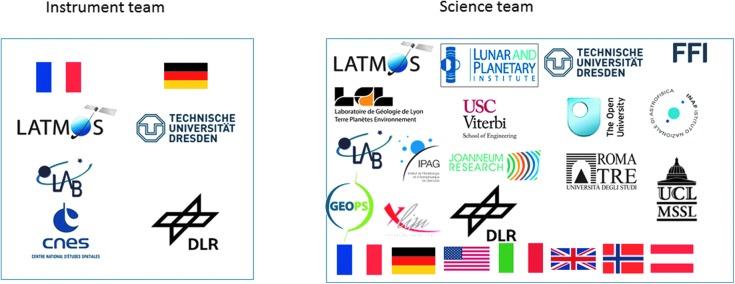
Nationalities and laboratory affiliations of the instrument and science teams.

The WISDOM radar is being developed through an international and multidisciplinary collaboration of science and technical teams (Fig. 1). The instrument is being built and tested by a French–German consortium supported by the French Space Agency Centre National d'Etudes Spatiales (CNES) and the German Space Agency Deutsche Forschungsanstalt für Luft-Und Raumfahrt (DLR) led by Valérie Ciarletti (Principal Investigator) from LATMOS (Laboratoire Atmosphères, Milieux, Observations Spatiales) in Guyancourt, France. LATMOS, in collaboration with Laboratoire d'Astrophysique de Bordeaux (LAB), is responsible for the electronics unit (EU) hardware manufacturing and delivery, while the Technische Universität Dresden (TUD, Germany) is responsible for the antenna system, under the supervision of Dirk Plettemeier (Coprincipal Investigator).

The WISDOM team consists of scientists from Europe (France, Germany, Italy, Norway, Austria, and United Kingdom) and the United States, who have a broad range of expertise in martian geology, geomorphology, hydrology, mineralogy, signal and image processing, electromagnetic (EM) modeling, and 3D visualization, which represents the best combination of skills necessary to analyze and interpret the WISDOM data. The team is led by the Principal Investigator, with the assistance of the Deputy Science Team Leader, Stephen Clifford, from the Lunar and Planetary Institute (LPI) in Houston, TX. To prepare for analysis of the ExoMars radar data, the team has conducted (1) field tests with a WISDOM prototype in various martian analogue environments, (2) dielectric characterization of analogue samples in the laboratory, and (3) numerical modeling of EM wave propagation in the subsurface, which takes into account the characteristics of the martian environment, as well as WISDOM operational and instrumental characteristics. These activities are essential to ensure the accurate interpretation of WISDOM data that will eventually be returned from Mars.

## 2. Science objectives

### 2.1. The ExoMars mission's science objectives

The scientific objectives of the ExoMars Rover mission (Vago *et al*., 2017) include the following: (1) search for evidence of past and present life; (2) investigate the water/geochemical environment as a function of depth in the shallow subsurface; and (3) investigate the planet's surface and subsurface to better understand the geologic and climatic evolution of Mars and their implications for habitability. To address these objectives, the rover is equipped with a powerful suite of instruments and tools, including a shallow (∼2 m) drill and the Pasteur science payload, which consists of a combination of survey, contact, and analytical instruments, all of which will be applied to the identification, acquisition, and analysis of surface and subsurface samples.

Current martian surface conditions of extreme dryness, low temperature, and continuous exposure to atmospheric oxidants and solar UV and cosmic rays are hostile to life as we know it. The predominance of such conditions throughout most of martian geologic history would make the survival of even the hardiest terrestrial microorganisms difficult on the surface. For this reason, the most likely place to find evidence of past or present life on Mars is underground.

Of all of the instruments in the Pasteur payload, only two—the WISDOM ground-penetrating radar (GPR) and the ADRON neutron spectrometer—have the ability to characterize the nature of the subsurface remotely and enable the identification of potential past habitats and environments where life, or evidence of its previous existence, may be preserved.

### 2.2. WISDOM science objectives

To achieve the Rover mission's science objectives, the WISDOM GPR will conduct high-resolution investigations of the shallow subsurface and address the following science objectives:

• Understand the 3D geology and geologic evolution of the landing site, including lithology, stratigraphy, and structure; the scale and magnitude of spatial heterogeneity; and the EM, physical, and compositional properties of the subsurface.
• Investigate the local distribution and state of subsurface H_2_O, including the potential presence of ice-rich frozen ground, segregated bodies of ground ice, and the persistent or transient occurrence of liquid water/brine.
• Identify the most promising targets for subsurface sampling as well as the potential hazards such as the presence of buried rocks, which could damage the drill and jeopardize the successful retrieval of samples.

Geological processes (*e.g.,* aeolian, volcanic, impact, fluvial, lacustrine, and periglacial) often produce deposits whose stratigraphy, bedding geometry, fracture orientation, and embedded block size are diagnostic of their origin and timing. Terrestrial field investigations, with rover-deployable GPR prototypes, have demonstrated that a GPR can aid in the identification and determination of the orientation and spatial variability of the above features (*e.g.,* Annan and Davis, 1976; Arcone *et al*., 1998; Hinkel *et al*., 2001; Yoshikawa *et al*., 2004). Our team's preparation work has contributed to a growing database of GPR observations that can be used to help establish the geologic setting and history of the rover environment and select the locations of greatest interest for further investigation (*e.g.,* Grant *et al*., 2004; Grimm *et al*., 2006; Heggy *et al*., 2006a; 2006b; Ciarletti *et al*., 2011; Russell *et al*., 2013; Dorizon *et al*., 2016). This terrestrial experience provides additional confidence in our ability to accurately analyze and interpret the WISDOM data to identify geologic processes characteristic of potentially past habitable environments (Jol and Smith, 1991; Havholm *et al*., 2003; Lipinski, 2009; Knoph, 2011).

On Mars, evidence of past or present life may be found in subsurface environments associated with the past or present occurrence of liquid water or in environments in which its biochemical signature may be preserved in massive ground ice or aqueous sediments. Knowing the abundance, distribution, and state of subsurface water (both as a liquid and as ice) is key for understanding the evolution of the martian surface, atmosphere, and climate, as well as the potential for the origin and continued survival of life. It is also useful as an *in situ* resource for sustaining future human explorers.

#### 2.2.1. Liquid water

During the summer, at low latitudes (and at mid-latitudes in regions of low albedo and large equatorward-facing slopes), the temperature of the top few centimeters of the regolith can exceed 273 K for as much as several hours a day, potentially melting frost and/or near-surface ice. The presence of potent freezing-point depressing salts (such as calcium chloride, magnesium, and calcium perchlorate) could conceivably extend the duration of melting to lower temperatures, allowing the survival of local brines throughout the hemispheric spring and summer (Brass, 1980; Clark and Van Hart, 1981; Knauth and Burt, 2002; Hecht *et al*., 2009; Martinez and Renno, 2013). Indeed, this has been proposed as a possible mechanism for the origin of recurrent slope lineae (RSL), dark streaks that develop in spring on equatorward-facing slopes at low to mid-latitudes, which appear to propagate downhill on a timescale of weeks to months and then fade away by the end of summer (McEwen *et al*., 2011, 2014; Grimm *et al*., 2014; Stillman *et al*., 2014; Ojha *et al*., 2015). The origin of the water involved in RSL activity is still unclear, with hypotheses ranging from the melting of subsurface ice, the discharge of a shallow aquifer, or the deliquescence of regolith salts by atmospheric water vapor (Ojha *et al*., 2015).

GPR excels at detecting liquid water and brine because of their high permittivity values relative to dry rock, soil, and ice. For a GPR, the identification of transient liquid water or brine in the near subsurface is possible by observing diurnal or seasonal variations in surface permittivity at the same location. Although the unambiguous identification of liquid water may be complicated by the temperature-dependent dielectric and magnetic properties of some martian minerals (Stillman and Olhoeft, 2006), the dielectric contrast of liquid water relative to that of frozen ground is sufficiently great that at concentrations as small as a few percent, its presence should be detectable. However, gradients in liquid water content, such as those present in the capillary fringe above a water table or as thin films of supercooled water present in temperate permafrost (Grimm *et al*., 2006; Le Gall *et al*., 2007), can also reduce the dielectric contrast between materials and make it difficult to identify transitions between differing lithologic and volatile materials.

#### 2.2.2. Water ice

A record of past biological activity may also be preserved as organics in subsurface ice and aqueous sediments. On Mars, massive ice may occur as the buried frozen remnant of a former lake or outflow channel discharge, where organic molecules, microbial fossils, and relatively unmodified samples of the aqueous geochemistry of subpermafrost groundwater might still be preserved.

The dielectric properties of ground ice and high porosity sediment are similar, making them difficult to differentiate based on radar measurements alone. However, where there is independent observational evidence of the presence of ice (whether by direct observation, neutron spectroscopy, or by the presence of geomorphic indicators, such as ice wedges and polygonal ground), a GPR can determine subsurface structure, density, and dielectric properties from which plausible ranges of ice concentration can be deduced. Although GPR cannot reliably determine the amount of ice present when its volume fraction is small compared with that of the regolith, it can be used to readily quantify and map the distribution of ice when it is present as segregated ice in units with dimensions larger than tens of centimeters. In this way, GPR could map the distribution of near-surface ice in latitude-dependent mantles, where it is believed to persist to depths significantly greater than those accessible by neutron spectroscopy (which is limited to ∼1 m).

#### 2.2.3. Former aqueous environments

A GPR can also identify geometric and geoelectric evidence of former aqueous environments in the shallow subsurface, such as the scale and orientation of layers and sediments, buried channels, shorelines, and erosional unconformities. GPR excels at mapping subsurface structures, such as the changing thickness and extent of beds that relate to depositional settings (*e.g.,* lacustrine vs. fluvial). The radar contrast between deposits in these environments has yielded fundamental information about the location, depositional/environmental context, and duration of paleolakes and paleochannels on Earth (Wilkins and Clement, 2007; Bridge, 2009).

For example, Gale Crater, the NASA Curiosity Rover landing site, contains at least one example of an ancient habitable lake environment (Grotzinger *et al*., 2014, 2015) that was subsequently modified by a variety of geologic processes with complex timing relationships. Superimposed structures range from aeolian sand drifts and their lithified counterparts to mudstones and sulfate veins. Similarly, the Spirit and Opportunity Mars Exploration Rovers (MERs) have both encountered evidence of ancient, potentially habitable aqueous environments that may be similar to those that are believed to exist at Oxia Planum, one of the ExoMars candidate landing sites. For example, Spirit's exploration of silica-rich deposits and carbonate-bearing outcrops in Meridiani Planum found evidence for fluvial activity and clement surface conditions at the time of their formation (Squyres *et al*., 2008; Morris *et al*., 2010), while Opportunity's exploration of the rim of Endeavour Crater (Squyres *et al*., 2012; Arvidson *et al*., 2014) has provided insights into the nature of impact-generated hydrothermal systems, which have been identified as an important source for habitability on early Mars (Rathbun and Squyres, 2002; Schwenzer and Kring, 2009).

WISDOM can also identify the dielectric properties and vertical distribution of rocks and soil whose attenuation characteristics are consistent with the presence of salts and clays, which represent potential evidence of aqueous alteration by the former presence of liquid water or brine. In places where independent compositional measurements performed by other instruments of the Pasteur payload have identified the presence of salts or clays, the radar attenuation characteristics of the regolith will help to determine the distribution of these materials as a function of depth—at least up to the maximum sounding depth of WISDOM. This provides a relative measure of the duration of the environmental conditions that gave rise to these materials.

In summary, the WISDOM data will aid our understanding of the geologic context of the landing site and Rover positioning, including (1) the local lithology, stratigraphy, and structure; (2) the role of water in the local sediment cycle (identifying the extent and duration of prior flowing and standing bodies of water (*e.g.,* rivers and lakes); and (3) areas where liquid water (including brines) may have existed at or near the surface in a relatively recent past. This is critical for the 3D understanding of the site and identification of the most scientifically interesting and safest places for ExoMars to drill.

### 2.3. WISDOM at Oxia Planum

Oxia Planum was chosen as the prime candidate landing site for the ExoMars Rover mission because it best satisfies the various rover engineering constraints and science requirements, which include the following: (1) the site must be ancient (>3.6 Ga), dating from the time when martian conditions are thought to be the most favorable to the emergence of life; (2) the site must possess abundant morphological and mineralogical evidence for long duration or reoccurring aqueous activity; and (3) the site must include widespread access to sedimentary rocks, throughout the landing ellipse, to ensure that they are accessible by the rover (ExoMars 2018 Landing Site Proposal Guide, 2013). Oxia Planum has an extensive clay-bearing unit dated at 3.9 Gy (Quantin *et al*., 2016). Phyllosilicates, identified by their diagnostic absorptions at ∼1.4, ∼1.9, and ∼2.3 μm, are exposed over about 80% of the landing ellipse (Carter *et al*., 2016) in a light-toned unit that exhibits significant layering, down to the ∼30 cm resolution limit of the HiRISE (High-Resolution Imaging System) digital terrain model. The analysis of the stratigraphy exposed in crater walls shows several well-identified layers in the top 10 m, with individual thickness ranging from 0.4 to 5 m (Ciarletti *et al*., 2015). Fluvial morphologies, such as valleys, inverted channels, fans, and deltaic deposits, are observed within the landing site (Quantin *et al*., 2016). On flat surfaces, the clay-rich layered formation exposes polygonal features that suggest a record of either past periglacial conditions or desiccation processes. Thus, WISDOM can expect to find a diverse geologic environment that includes fine and complex layering, cross bedding, buried channels, and desiccation wedges.

### 2.4. Synergies with other instruments of the Pasteur payload

The best instrumental complements to WISDOM, within the Pasteur payload, are PanCam, the Rover's mast-mounted Panoramic Camera (PanCam), and the neutron spectrometer ADRON.

PanCam consists of two multispectral, wide-angle stereo cameras (with an instantaneous field of view (iFoV) of 652 μrad/px) and one high-resolution monoscopic color camera (iFoV = 83 μrad/px), which can zoom-in on near (<1 m) targets to infinity (Coates *et al*., 2017). This combination will provide a comprehensive understanding of the size and spatial distribution of blocks, outcrops, dunes, and other geologic features around the rover. WISDOM's goal is to provide an equivalent understanding (with a resolution of several centimeters over a few meters in depth) of the geology of the subsurface and the stratigraphy, structure, and distribution of embedded block along the rover traverse. Together, data from both instruments will help construct an improved picture of the geologic setting and evolution of the rover environment. This synthesis will be aided by PanCam's visual data representation tools, which will enable 3D visualizations of the PanCam and WISDOM data in the same coordinate frame. Preliminary, but promising, demonstrations of this capability (Paar *et al*., 2015) were obtained during the ESA-organized Sample Acquisition Field Experiment with a rover (SAFER) experiment that took place in 2013 in the Atacama Desert, Chile (Gunes-Lasnet *et al*., 2014).

The neutron spectrometer ADRON (Mitrofanov *et al*., 2017) is the only Pasteur instrument aside from WISDOM that is capable of investigating the composition of the subsurface before drilling. During the rover traverse, ADRON will provide the averaged bulk content of hydrogen within the first meter, providing information about the hydration state and distribution of water in the shallow subsurface. At the same time, WISDOM will obtain information about the stratigraphy and structure over a depth ranging from 3 to 10 m of the regolith. During each experiment cycle (EC) of the mission, the rover will execute a subsurface scanning pattern with both WISDOM and ADRON with the objective of identifying a suitable place to drill. The interpretation of data from each instrument will benefit from others' findings. For example, WISDOM's information about the stratigraphy of the shallow subsurface will aid ADRON's interpretation of the areal and vertical distribution of water. In return, ADRON data will help constrain the identification of geological units and potential state of water (*e.g.,* liquid or ice) detected by WISDOM. A joint, prelaunch field test is planned in a permafrost environment in Siberia to help optimize the exchange of data and further enhance the operational synergies of both instruments when they arrive on Mars.

Only one instrument onboard the rover will have direct access to the subsurface, that is, the Mars Multispectral Imager for Subsurface Studies (Ma_MISS) spectrometer (De Sanctis *et al*., 2017), which will be accommodated inside the drill and will study subsurface stratigraphy and geochemistry *in situ* while the drill is operating. During each EC, and especially during the few vertical surveys (VS; down to 2 m), the subsurface stratigraphy detected by WISDOM will be compared with, and interpreted within, the context of compositional variations observed with depth by Ma_MISS. WISDOM, in turn, will be able to extrapolate the local information provided by the spectrometer into two-dimensional (2D) or 3D subsurface maps.

Finally, during the course of the ExoMars mission, the samples retrieved by the drill will be analyzed by the rover's suite of exobiology and geochemistry instruments. They will provide information about the physical and chemical properties of the subsurface that will provide ground truth for interpretation of the WISDOM data. From these observations, the EM characteristics of the materials may then be inferred with reasonable accuracy through comparisons with existing laboratory measurements of EM properties of Mars analogue materials. This knowledge should significantly improve the accuracy and quantitative interpretation of the WISDOM data. We can expect that as the mission progresses, the repeated comparison of WISDOM and ADRON data with the ground-truth compositional and physical property information provided by other instruments (based on the analysis of borehole mineralogy and collected samples) will allow our team to improve its ability to infer and interpret subsurface properties.

The suite of instruments on the 2020 ExoMars Rover will provide a comprehensive set of data regarding the geology and geologic history of the landing site that will aid in the selection of the best locations and depths to drill and sample. WISDOM will assist this process by providing information on the stratigraphy and structure of the subsurface, including the potential presence of subsurface water and ice.

## 3. Instrument Design and Anticipated Performances

In agreement with the science objectives described above, WISDOM was designed to investigate the dielectric properties and structure of the near subsurface, down to a depth of ∼2–3 m, with a vertical resolution of a few centimeters.

### 3.1. Instrument description

WISDOM is a step frequency (SF) radar that operates in the frequency domain. For each sounding, a succession of *N* harmonic pulses of duration τ that sweep the instrument's frequency bandwidth *B* are transmitted through the surface and reflected by changes in permittivity in the subsurface. An inverse Fourier transform (IFT) is then performed on the data to retrieve the time-domain impulse response of the surface and subsurface echoes. An image is eventually built from a number of soundings that show variations in permittivity within the subsurface. The SF design allows for high resolution in the time domain with a low electronics noise level due to the narrow instantaneous bandwidth ${B_{RX}}$ of the receiver.

The instrument range (*i.e.,* distance) resolution $\Delta r = c / 2B$ is determined by the frequency bandwidth and the velocity *c* of the EM wave inside the medium, while the value of the frequency step $\Delta   f = B/ N$ determines the nonambiguous range (a target located at a distance larger than the nonambiguous range would be interpreted to be at an apparent much closer distance due to range folding). The frequency range from 0.5 to 3 GHz was selected for WISDOM to provide a sounding depth of a few meters in a lithic environment (commensurate with the maximum depth that can be reached by the drill) and a vertical resolution of a few centimeters. WISDOM's nominal operational parameters values are listed in Table 1. These values result in a vertical resolution of ∼3 cm and a maximum depth of sounding of 12.5 m for a typical dielectric constant value of 4.5 (in a homogeneous and lossless environment).

**Table 1.** Main WISDOM Instrumental Parameters

		*Typical values*
Center frequency	*f_0_*	1.75 GHz
Frequency bandwidth	*B*	2.5 GHz
Wavelength in vacuum	λ	0.1–0.6 m
Number of frequency steps	${ \rm{N}}$	1001
Frequency step	$\Delta { \rm{f}}= B / N$	2.5 MHz
Step duration	τ	200 μs
Transmitted power	*P_T_*	0 dBm
Noise factor	*NF*	8 dB
Receiver frequency bandwidth	${B_{RX}}$	1 kHz
Receiving channel adjustable gain	${G_{RX}}$	−7 to 25 dB
Antenna Gain	${G_{aTX}} = {G_{aRX}}$	1 to 8 dB
No. of ADC bits	${N_{ADC}}$	16
Effective dynamic range of the ADC	*DR*	∼84 dB
Number of coherent additions	${N_{add}}$	10
Receiver and transmitter efficiency	${e_{TX}} = {e_{RX}}$	0.1
IFT gain	${{ \rm{G}}_{IFT}}$	27 dB
Coherent additions Gain for 10 additions	${{ \rm{G}}_{add}}$	10 dB

ADC = analog-to-digital converter; IFT = inverse Fourier transform; WISDOM = Water Ice Subsurface Deposit Observation on Mars.

Because of its SF design, WISDOM has no blind zone, so it also receives the surface echo that can be used to get an estimate of the permittivity of the near subsurface (Sect. 3.4).

The WISDOM instrument consists of two main subsystems: the EU and the Antennas System, which are described below [for greater detail, see Ciarletti *et al*. (2011)].

### 3.2. Description of the EU

The EU consists of three separate boards that correspond to the instrument's three main subsystems: the radio frequency module, the digital module, and the DC/DC convertor module. Each has been designed to minimize the EM interferences inside the EU and with other rover instruments and equipment.

Under nominal conditions, WISDOM is expected to receive its first strong echo from the surface, followed by other weaker echoes from progressively deeper structures and interfaces within the subsurface. The instantaneous DR (dynamic range), which is the ratio between the strongest and weakest signal that can be measured with the same instrument settings, is one of the most important characteristics of the instrument. It is limited by the number ${N_{ADC}}$ of bits of the analog-to-digital converter (ADC). The maximum DR (in dB) is given by the following formula:

\begin{align*}
DR = 20\;{ \rm{*}}\;log10 \left( {{2^{{N_{ADC}}}}} \right) . \tag{1}
\end{align*}

This theoretical value is never reached because errors reduce the number of effective bits. For the 16-bit ADC used by WISDOM, the effective DR is about 84 dB (compared with the theoretical value of 96 dB). It is the effective dynamic that determines the ability of the instrument to detect weak echoes.

Adjustable attenuators are used in the EU to avoid saturation by the strongest echo, but then weaker echoes are equally attenuated and can be missed. Therefore, to make the best use of ADC dynamics, hard gating can be used to delay reception by an interval long enough to avoid the strongest echo coming from the surface and thus focus on the delayed and weaker echoes coming from deeper structures/interfaces in the subsurface. Hard gating in WISDOM is optional and will only be used if the effective dynamic of the instrument is not sufficient to get the whole picture at once. The soundings performed when using hard gating are complementary to those performed without. In this way, the full DR range can be used to detect the surface echo as well as the delayed and weaker echoes.

The design chosen for the WISDOM instrument offers good versatility, in that it allows us to adjust the instrument's operating parameters (such as the number of steps, the attenuation values in the receiving and transmitting chains, and gating parameters) to optimize its performance for the actual conditions found on Mars.

### 3.3. Description of antenna unit

WISDOM employs two similar dedicated antennas to transmit and receive signals over a very large frequency bandwidth. They will be mounted next to each other at the rear of the rover, where the interaction with the rover body and wheels will be minimized. To enhance the scientific return of WISDOM data, polarimetric soundings are planned. As a consequence, each antenna has been designed to allow transmission or reception with two perpendicular polarizations. By comparing the characteristics of the reflected waves and using each of the four possible copolarization and cross-polarization alignment pairs, details of the location, orientation, and shape of the reflector can be determined. This improves WISDOM's ability to retrieve the geometrical and physical properties of buried rocks, reducing the left/right ambiguity normally associated with determining the location of off-axis reflectors, and aids in determination of the geometry and roughness of structural or stratigraphic interfaces, attributes that are crucial to understanding the geological processes that formed them.

The WISDOM antennas have been designed to meet the requirements summarized in Table 2.

**Table 2.** Requirements of the WISDOM Radar Antenna

Frequency range	0.5 to 3.0 GHz
Polarization	Two perpendicular polarizations for transmitting and receiving antenna
Reflection coefficient on a 50-Ω-system	≤ −10 dB
Radiation pattern	homogeneous main lobe
Half-power beamwidth	Θ > 20° and Θ_H-plane_ > Θ_E-plane_
Gain in main beam direction	>0 dBi increasing with frequency
Cross-polarization ratio	< −15 dB
Mass	400 g
Structural	Withstand mechanical loads during vibration and shock

The basic antenna element is designed as a tapered slot antenna, where the taper follows an exponential function. This kind of tapered slot is known as a Vivaldi antenna and is very broadband with respect to its reflection loss at the antenna ports and its gain function. The lower frequency bound of the antenna depends on the length of the tapered slot and maximum size of the aperture, which is constrained by the size of the antennas that can be accommodated on the rover. Over the instrument's bandwidth, the gain of the antenna increases with frequency, which benefits GPR applications since propagation losses in the subsurface also increase with frequency.

The electrically conductive antenna structures are etched on a thin, planar copper-cladded substrate. This is the base for a lightweight design, which is necessary to stay within the allowed mass budget for the whole instrument. The radiation pattern of the planar structure is symmetric both in the E-plane (which is the plane of antenna element) and in the H-plane (which is perpendicular to the antenna element).

**FIG. 2.**
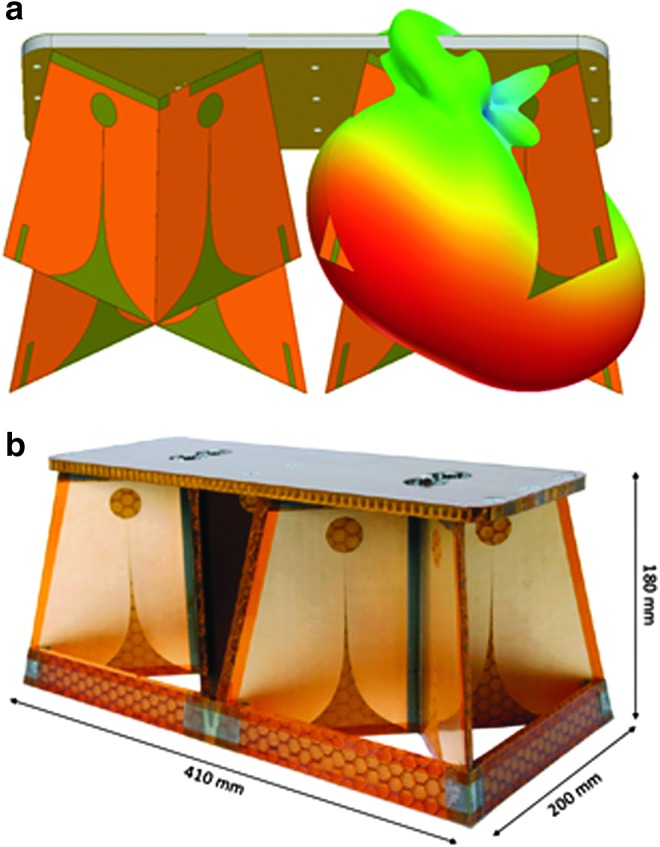
Antenna arrangement and gain pattern at 2 GHz, **(a)** and side view of the structural thermal model of the antenna **(b)**.

As mentioned above, the radar is capable of transmitting and receiving in two orthogonal linear polarizations, which yield four transfer functions of the ground target. However, in practice, there are some design challenges, especially for the feeding and for the slot because its width is smaller than the thickness of the substrate. To resolve this issue, two Vivaldi tapered slot antennas are arranged on each of the two orthogonal radiating elements (Plettemeier *et al*., 2009) so that their intersection lies between the two slots (Fig. 2). If this is done properly, the influence of one element on the field of the other is minimal and both polarizations will have the same center of phase. Moreover, due to this configuration, the length of the slots can be reduced, which increases the first mechanical resonance frequency, thus allowing some mass saving, because the construction of the surrounding support structure can be lighter. As a consequence of this innovative design, the transmitted signal must be divided (or combined at the receiver), which is done by a Wilkinson divider/combiner (Benedix *et al*., 2013).

The two crossed antenna elements are glued to a common base plate through brackets, which are strengthened by a frame of stiffeners to increase the first mechanical resonance frequency for vibration and shock loads. The crossed antenna elements are rotated by 45 degrees with respect to the Rover path (Fig. 2) not only to save space but also to detect whether scattering objects in the subsurface are located to the left or right of the rover. Both the surrounding support structure and the substrate for the antenna elements are made of a single type of honeycomb glass fiber reinforced plastic. The baseplate is also honeycomb, but is made of aluminum for its increased stiffness. The conductor used for the Vivaldi antennas is a thin layer of copper, the surface of which is treated with a gold layer. The baseplate accommodates both the mechanical and electrical interfaces to the rover. All materials used are listed in the outgassing databases of ESA and NASA and are used in other space applications, such as the TROPOspheric Monitoring Instrument spectrometer on the ESA Sentinel-5 Precursor satellite (Veefkind *et al*., 2012). The antennas are designed to withstand a dry heat microbial reduction to be compliant with a planetary protection category IVb, which is requested for the mission.

Unlike most terrestrial GPR antennas, the WISDOM antenna assembly is mounted on the rover with a clearance between aperture and ground of about 30 cm. This accommodation, while improving rover obstacle clearance, weakens the strength of the signal transmitted into the ground. In addition, the gap between the ground and rover structure may cause multiple reflections. To suppress this kind of ringing, the antenna is designed to have a low radar cross section and high forward-to-backward gain ratio.

### 3.4. Performances of the instrument

A simplified analytical approach allows us to obtain rough estimates of the performance of WISDOM for some typical configurations. We focus here on the detection depth (depth at which we can detect a target buried within the local environment) and the range resolution achieved under the same conditions. In our case, the signal-to-noise ratio (SNR) is the key parameter, which is given by

\begin{align*}
SNR = { \frac { { P_R } }  { { P_N } } } \tag { 2 } 
\end{align*}

where *P_R_* is the power of the signal reflected by a buried target and *P_N_* is the power of the noise at the receiver.

A detection usually requires an SNR value larger than 3 dB. However, a quantitative analysis of the signal (sufficient to yield a good estimate of the permittivity) would require a larger SNR (typically 10 dB). Note that *P_N_* is proportional to the instantaneous bandwidth ${B_{RX}}$ at the receiver:

\begin{align*}
{P_N} = NF. \ {B_{RX}} \tag{3}
\end{align*}

The useful signal power *P_R_* for a given frequency can be estimated with the radar Eq. 4, which takes into account the main instrument parameters given in Table 1 and some additional parameters that describe the geometrical configuration, target, and medium characteristics.

\begin{align*}
 { P_R } = { P_T } { e_ { TX } } { G_ { aTX } } \ { e_ { RX } } { G_ { aRX } } { \frac { { \lambda ^2 } }  { { { \left( { 4 { { \pi } } } \right) } ^3 } } } \frac { 1 }  { { { d^4 } } } \ { A_ { Att } } { A_ { Sc } } { S_T } \tag { 4 } 
\end{align*}

where *d* is the distance between the target and the transmitting and receiving antennas (assumed to be at the same location for the purpose of this simplified study), ${G_{aTX}}$ and ${G_{aRX}}$ are the transmitting and receiving antenna gains, ${e_{TX}}$ and ${e_{RX}}$ are the transmitter and receiver channel efficiencies, *S_T_* is the target radar cross section (depending on the size, roughness, and dielectric contrast of the target and on the frequency), and ${A_{Att}}$ is attenuation in the medium due to electrical losses, which include polarization and conduction phenomena.

In the case of a low-loss medium, ${ A_ { Att } } = { { \rm { e } } ^ { \textstyle - { \frac { 2 \pi { \varepsilon { \prime\prime } } }  { \lambda \sqrt { \varepsilon \prime } } } 2d } }$ where ${{ \varepsilon \prime}}$ and ${ \varepsilon{\prime\prime}}$ are the real and imaginary components of the effective relative permittivity of the medium. Typical values for some martian analogue materials are given in Table 3 (see Section 5.1 for laboratory measurements). The effective conductivity $\sigma = 2 \pi f{ \varepsilon _0}{ \varepsilon{\prime\prime}}$ is often used to characterize these losses. ${A_{Sc}}$ represents the losses due to wave scattering and is also a function of the frequency. The scattering phenomenon is negligible if the size of the heterogeneities inside the medium is much smaller than the wavelength (10 cm for WISDOM's highest frequency in vacuum). In our simplified approach, this effect is neglected, but will be considered in the numerical simulations presented in Section 5.

**Table 3.** Permittivity and Permeability Values Measured for Different Martian Soil Simulants (Heggy *et al*., 2001; Heggy *et al*., 2003; Pettinelli *et al*., 2007; ElShafie and Heggy, 2013)

	*Porosity (%)*	*450–1200 MHz*			
Materials		ɛ′	ɛ″	μ′	μ″
Water ice (−20°C) (∼400 MHz)	0	3.10	0.03	1.0	—
Liquid water (25°C) (∼400 MHz)	0	79.26	1.7	1.0	—
Dry sand deposit	∼30	2–3 ± 0.02	0.01–0.001	—	—
Unconsolidated sand	50	3.03 ± 0.02	0.0019–0.0013	1.01 ± 0.01	<0.001
Dry sandstones	∼30	3–5 ± 0.01	0.03–0.001	—	—
Magnetite 10% 200–500 μm (20°C)	37.5	4.2 ± 0.06	0.0003–0.0001	1.22 ± 0.07	0.003 ± 0.002
Dry Limestone	∼30	4–5 ± 0.02	0.2–0.002	—	—
Permafrost with 38% water (−28°C)	28	5.34 ± 0.04	0.06–0.002	1.0	—
Tephra deposits (14% FeO)	38	5–3 ± 0.02	0.15–0.01	1.1 ± 0.03	<0.001
Basalt (20°C)	18	6.2 ± 0.2	0.0008–0.0005	1.00	—
Dry clay	∼30	6–9 ± 0.02	0.3–0.02	—	—
Low-porosity lava flow	14	7–5 ± 0.04	0.3–0.2	1.2 ± 0.04	<0.002

Equation 4 demonstrates that the received power decreases with the depth of the target, higher frequency, and greater subsurface conductivity. As explained before, WISDOM collects a data set of ${ \rm{N}}$ measurements, performed at different frequencies, to retrieve the desired response in the time domain, using an IFT. As a consequence, it is the SNR obtained in the time domain that is used for data interpretation.

For a given ${A_{Att}}$ and *S_T_*, we use Eq. 4—accounting for the gain resulting from the number of coherent additions and the IFT—to identify, for a given target depth, the frequencies that will significantly contribute to the target detection. The achievable range resolution for the considered depth is estimated from the frequency bandwidth actually received by the instrument.

To illustrate the performance capabilities of WISDOM, we consider two kinds of targets: a smooth interface and a moderately rough one, for which the area contributing to the reflection is assumed to be limited to the first Fresnel zone (Cook, 1975; Grimm *et al*., 2006). The layer overlying these interfaces is characterized by its complex effective relative permittivity. For the real part ${{ \varepsilon \prime}}$, we assume a value of 6, which is intermediate between clay and basalt (Table 3). For dielectric losses, we consider three different values of ${ \varepsilon{\prime\prime}}$: (1) the highest, ${ \varepsilon{\prime\prime}} = 0.25$, is representative of dry clays or limestone and low-porosity lava and is consistent with the values estimated from the data collected by the SHARAD radar sounder (on the Mars Reconnaissance Obiter) over the northern plains of Mars (Campbell *et al*., 2008; Stillman and Grimm, 2011); (2) a significantly lower value, ${ \varepsilon{\prime\prime}} = 0.01$, corresponds to dry sand deposit; and (3) an intermediate value, ${ \varepsilon{\prime\prime}} = 0.1$, is representative of permafrost.

**FIG. 3.**
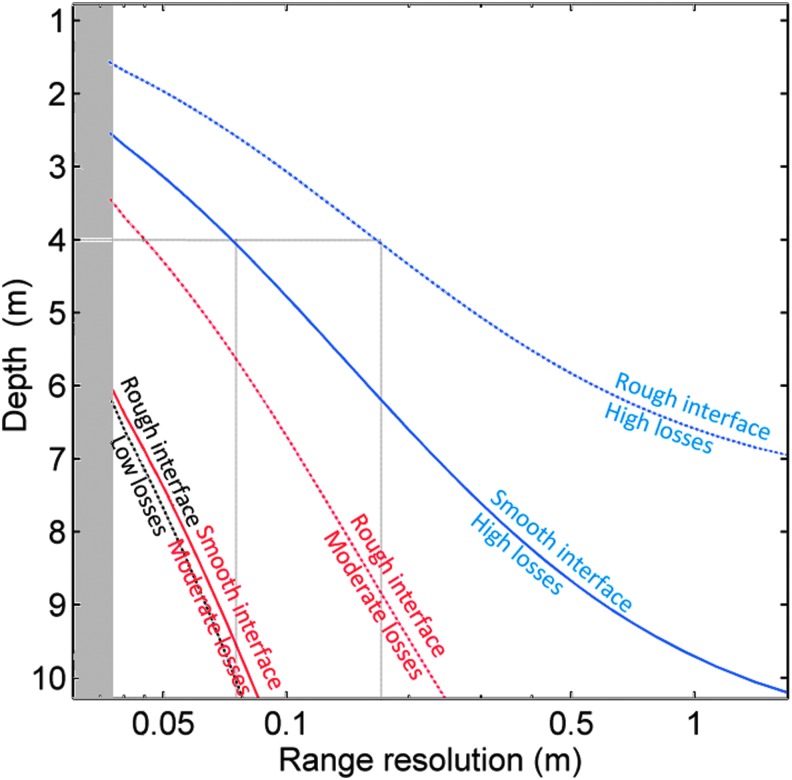
Estimated range resolution and depth for a rough (dotted curves) or a smooth (solid curves) interface with three different electrical losses corresponding to ɛ″ = 0.05 (black); ɛ″ = 0.1 (red); and ɛ″ = 0.25 (blue). The gray area corresponds to range resolutions better than 3 cm that cannot be achieved with the WISDOM frequency bandwidth. WISDOM, Water Ice Subsurface Deposit Observation on Mars.

Figure 3 shows the results of simulations obtained for an SNR > 3, in terms of range resolution and depth, for the combination of interfaces and permittivity values considered. This modeling takes into account the key parameters of the WISDOM radar and provides rough estimates of the detection depth and associated range resolution for a few typical subsurface configurations. The scattering encountered by EM waves traveling through nonhomogeneous layers has been neglected.

The best range resolution (3 cm) is achieved when the reflected signal from the interface is received, with an SNR >3 dB, over the entire 0.5–3 GHz operational frequency range of WISDOM. As the conditions become less favorable (larger depth, higher losses, or rougher target), the higher frequencies are attenuated and the resolution decays. For example, at a depth of 4 m in a high-loss environment (as highlighted by the gray lines on the figure), a rough interface would be detected with a vertical resolution of ∼18 cm, while the vertical resolution would be ∼7 cm for a smooth interface. The lower the loss and the smoother the target interface, the better the resolution that can be achieved up to the theoretical maximum of ∼3 cm with WISDOM's 2.5 GHz bandwidth.

## 4. Measurement Scenario

To maximize the science return of the ExoMars payload, a reference surface mission (RSM) has been studied and defined. The RSM includes six ECs and two VSs. During each EC, all payload instruments will be operated and two samples will be collected (one at the surface and one in the subsurface). The total surface travel is ∼1.5 km. WISDOM is one of four panoramic instruments (which also include the PanCam, Infrared Spectrometer for ExoMars [ISEM], and neutron detector [ADRON]) that will be used to characterize the geologic context of the rover and the selection of optimum locations for surface and subsurface sampling. Achieving these goals requires investigations at different spatial scales that are only possible because of the instrument's flexible and programmable settings and design.

### 4.1. How will the instrument be used?

Terrestrial experience has demonstrated that 3D techniques can significantly reduce the interpretive ambiguities associated with identifying subsurface structures and discriminating between lithologic and volatile units. WISDOM will contribute to understanding the 3D geology of the landing site in two important ways:

(1) By conducting radar soundings at frequent intervals along the rover's traverse to construct 2D profiles of the subsurface over distances ranging from a few tens of meters up to several hundreds of meters, depending on the length of the EC. The along-track spatial resolution of such profiles is enhanced when the interval between successive soundings, which is controlled by navigation commands given to the rover, is reduced. In areas where the rover traverses cross or pass within close proximity of each other, the GPR sounding data can be used to provide a coarse 3D understanding of the local geological context, which can be used to investigate how the stratigraphy exposed in nearby outcrops and the interior of impact craters compares with the subsurface stratigraphy of the rover environment. Field tests, with a remote-controlled rover in terrestrial analogue environments, have shown that WISDOM can be operated continuously while the rover is in motion during a traverse (Gunes-Lasnet *et al*., 2014) at a reduced speed of around 20 m/h.

(2) By conducting targeted, high-resolution polarimetric surveys of areas of scientific interest, which are necessary for the identification and evaluation of potential sampling sites for the rover's drill. Such surveys (informally called a WISDOM grid) will be conducted by acquiring either three 10-m-long or five 5-m-long parallel profiles, ∼1 m apart, from which an interpolated 3D map of the local radar stratigraphy can be constructed. To detect buried rocks and make the best use of the polarimetric capability of the instrument, the spacing between consecutive soundings has been set to 10 cm, which is the maximum permissible distance for a correct 2D migration of data along track. At each stop, WISDOM will conduct four soundings that correspond to the four possible polarimetric combinations. According to the rover RSM, this kind of pattern will be executed for each EC, that is, at least six times during the nominal duration of the mission. Operation simulations on Earth, where WISDOM was mounted on a remote-controlled rover (Joudrier *et al*., 2012; Gunes-Lasnet *et al*., 2014; Dorizon *et al*., 2016), have proved that such results are achievable with the planned 10-cm along-track distance between soundings.

### 4.2. WISDOM ground data processing pipeline

#### 4.2.1. WISDOM data processing

With two communication passes per sol through the ExoMars orbiter, all the data necessary to plan the activities for the next sol need to be transmitted to the Rover Operations and Control Center (ROCC) during the evening pass and must be processed rapidly so that telecommands can be transmitted to the rover during the following morning pass.

All WISDOM data processing will be performed on Earth. The data volume for a WISDOM pattern is about 20 Mbits, which will be stored in a temporary memory onboard before being downloaded to Earth.

One key element in the WISDOM data analysis is the fast and reliable classification of geological features and correct localization of subsurface scatterers and layers. To get the best out of the instrument, data processing must be adapted to the sounded environment, but the elements remain the same. For each individual sounding performed in frequency domain, the data processing's major step is the transformation in time domain that can be rapidly obtained by an IFT. A window in frequency domain is needed to limit side lobes, and compensation for the instrument (antennas included) frequency response is applied. Once in time domain, the direct coupling between the antennas is the first echo detected; this provides a simple way of monitoring and compensating the instrument's potential drifts. Second comes the surface echo, which is analyzed to get a first estimate of the top layer's permittivity and surface roughness (Dorizon *et al*., 2016). Then, the median, which is computed on a number of soundings, is subtracted from the whole set of data to remove any instrumental artifacts that might occur at constant delay (such as reflections on a part of the rover structure or inside the instrument). A first image is built and available for a first global environment description. The measured delays can be converted into distances once permittivity of the medium is known. The permittivity at depth is estimated from radar signatures of embedded scatterers (if any). More sophisticated instrument-specific, tool-based migration techniques and compressed sensing have been developed (Lu *et al*., 2013; Zhang *et al*., 2013) and will be used in the future to process the WISDOM data.

A detailed analysis of the data over two or more frequency intervals that span the entire bandwidth allows for the study of frequency-dependent physical processes (scattering, losses) to better characterize the subsurface. The fully polarimetric nature of the WISDOM measurements allows the use of modern methods for polarimetric data processing and a better characterization of the shape and orientation of the reflecting structures.

#### 4.2.2. WISDOM data products

The different types of data products that will be delivered are shown in Table 4 with their estimated production/delivery time. The 2D radargrams converted to depth will be essential to plan the drilling operation. As a consequence, the time available for the processing is limited by the need to upload telecommands for operation during the next sol. These 2D radargrams will undergo progressive refinement between T1 + 45 min and T1 + 6 h (T1 being the time when the antenna system location and orientation are made available for the WISDOM team) to meet this deadline.

**Table 4.** WISDOM Data Products

*Product*	*Processing level*	*Description*	*Data production time*	
To: WISDOM data reception				
1	Telemetry	Telemetry data with data embedded	T0	
2	Raw	Raw frequency scans, mission time tagged	T0 + 15 min	
3	Partially Processed	Processed frequency scans, mission time tagged	T0 + 1 h	
4	Partially Processed	Bandwidth-specific processed frequency scans converted to travel time by IFT, mission time tagged.	T0 + 1 h	Rough description of the geological context
T1: Antenna's location reception				
5	Calibrated	2D radargrams vs. travel time; mission time converted to position and scans interpolated to produce uniform spacing Can be displayed in a 3D environment.	T1 + 30 min	Identification of *N* locations at the surface for drilling
6	Calibrated	2D radargrams with travel time converted to depth through uniform velocity or migration using a previously determined value of the dielectric constant value previously determined	T1 + 45 min	Rough estimate of the depths to collect a sample for the *N* locations
6bis	Calibrated	2D radargrams with travel time converted to depth through uniform velocity or migration using the dielectric constant value estimated from the current data set	T1 + 6 h	Best estimate of the depths to collect a sample for the *N* locations
7	Derived	Material properties (dielectric constants, attenuation coefficients)		
8	Derived	Interpreted 2D reconstructions		
9	Derived	3D reconstructions and interpretations		

2D = two-dimensional; 3D = three-dimensional.

**FIG. 4.**
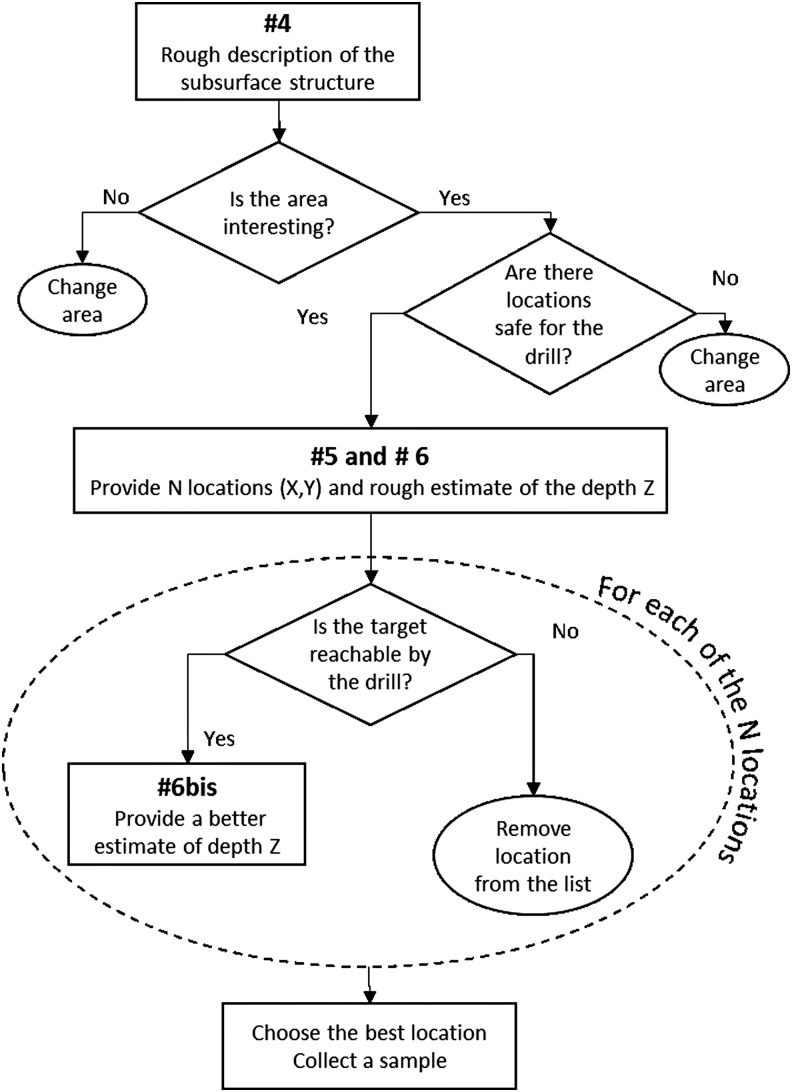
Selection process for the best location for the drilling operation based on WISDOM data.

The mission science team members will discuss the scientific interest of each area. WISDOM and ADRON's contribution to this discussion will be to provide information about the nature of the subsurface. For WISDOM, this will include a tentative identification of potential targets and estimate of their potential depth, which must lie within the maximum 2-m depth that can be achieved by the drill. Last, WISDOM can detect rocks that represent a potential hazard to the safe operation of the drill. This will be done by utilizing the rover's mobility to conduct radar soundings in a grid around the area of interest, supplemented by the boulder size, shape, and orientation information provided by WISDOM's polarimetric measurements. This will allow the team to determine the 3D location of the rocks and select a safe location (*i.e.,* minimizing the number of potential subsurface hazards–rocks) for drilling. If a large number of rocks are present, their signature will interfere, precluding the retrieval of individual locations. Given these conditions, the site would be considered unsafe for drilling. Figure 4 summarizes the safe drill site identification process based on the inputs from WISDOM. The numbers #4, #5, #6, and #6bis used in Fig. 4 are references to the data number in Table 4.

## 5. Data Interpretation

In a simple environment made of homogeneous units separated by smooth interfaces, detection of interfaces and retrieval of electrical properties are reasonably easy. In complex environments, wave propagation is modified by the roughness at interfaces and the volume scattering at spatial scales commensurate with the instrument wavelengths. As a result, quantitative interpretation of radargrams is not straightforward. This can only be efficiently achieved through three complementary tools and approaches that are currently being developed or adapted for use by WISDOM: (1) dielectric characterization of analogue samples in the laboratory, at ambient martian temperatures, over the frequency bandwidth employed by WISDOM for comparison with the future Mars data obtained by WISDOM; (2) numerical modeling of WISDOM radar propagation in realistic martian environments that will provide a data base of simulated radargrams that can be used to study the effect of subsurface characteristics (such as the size distribution of rocks, the roughness of interfaces) on the WISDOM data; and (3) field testing in a variety of martian analogue environments to validate WISDOM data processing techniques and interpretation as well as the instrument's end-to-end performance.

### 5.1. Dielectric characterization of samples in the laboratory

Laboratory characterization of the EM properties of martian analogue materials is critically important for (1) constraining the performance of the radar in different geological environments; (2) understanding the lithology and structure of the subsurface; (3) applying reliable inversion techniques to estimate the EM properties of deeper structures; and (4) evaluating the possible presence of liquid water in the subsurface.

The EM characterization of natural and artificial materials can be investigated in both the frequency and time domains. Both approaches are intimately connected and, theoretically, should yield the same results (Kao, 2004). From a practical point of view, however, frequency domain measurements are more complex than time domain methods. The main advantage of the frequency domain approach is that it allows computation of real and imaginary parts of permittivity and magnetic permeability of the sample from circuit parameters, assuming a specific equivalent circuit. In the time domain approach, the data analysis is more complicated and time-consuming. To obtain the same EM parameters, the time domain data should be transformed into frequency domain and fitted using some dielectric model (Chen *et al*., 2004; Kaatze, 2013).

At the frequencies of interest for WISDOM, the most suitable method with which to estimate the simulants' EM properties is based on the measurement of wave propagation parameters in a transmission line filled with test material. Velocity and attenuation of EM signals can be measured in the time domain by means of the so-called time domain reflectivity technique using a cable tester equipped with a pulse generator and an oscilloscope connected to the open end transmission line. The EM parameters are extracted from the travel time and amplitude of the signal propagating forward and backward in the probe (see, for details, *e.g.,* Ferré and Topp, 2000). In the frequency domain, the most common technique consists of a Vector Network Analyzer connected to a two-port transmission line; in this case, the real and imaginary parts of permittivity (and magnetic permeability) are estimated from the so-called scattering parameters (S-parameters), that is, the ratio of reflected or transmitted wave, at each port, to the incident wave (for details, see Chen *et al*., 2004).

Several studies have addressed laboratory determinations of the complex dielectric permittivity and the complex magnetic permeability of martian soil simulants (*e.g.,* Leuschen, 1999; Heggy *et al*., 2001, 2003; Pettinelli *et al*., 2003, 2005; Stillman and Olhoeft, 2004; Pettinelli *et al*., 2007; ElShafie and Heggy, 2013; Mattei *et al*., 2014). The values obtained in these works are difficult to compare as the measurements were performed on different samples (in terms of composition and texture) and at different frequencies and temperatures, which highlights the fact that special care must also be taken when interpreting permittivity values obtained from *in situ* radar measurements of similar materials. As an example, Table 3 shows the dielectric parameters' relative values measured on some of the geological materials collected in Earth dry analogs and expected to be found on Mars (Heggy *et al*., 2001, 2003; Pettinelli *et al*., 2007; ElShafie and Heggy, 2013). In the considered frequency range (450–1200 MHz), the real part of permittivity (ɛ′) and magnetic permeability (μ′) and the imaginary part of magnetic permeability (μ′′) are frequency-independent parameters, while dispersion of the imaginary part of permittivity (ɛ′′) for the same materials is illustrated. The data show that in these types of materials, WISDOM signal penetration should be at least of the order of several meters.

### 5.2. Numerical simulations on geological typical features

#### 5.2.1. Numerical modeling method

A 3D finite difference in time domain (FDTD) code called TEMSI-FD was developed by the XLIM (Limoges) to perform numerical simulations of radar operations in realistic environments (Martinat, 2001; Heggy *et al*., 2003). The FDTD method is based on the algorithm of Yee (1966); the medium is discretized in volume elements (Yee's cells) in which Maxwell's equations are solved by a time-stepping procedure, thus providing the spatial and temporal variations of the EM field in the medium. We use this code to test WISDOM performances over various environments.

The advantage of the FDTD method compared with an analytical approach relies on its accuracy and versatility, which largely counterbalances the concomitant increase of computation time. To avoid numerical dispersion, the size of discrete volume elements (Δ*x*, Δ*y*, Δ*z*) must be at least of the order of λ/10 where λ is the wavelength in the most refractive medium for the highest frequency of operation (3 GHz). In practice, the spatial step was set to 5 mm in three perpendicular directions. Furthermore, the time step Δ*t* must remain smaller than the wave propagation time in a cell to satisfy the Courant–Friedrichs–Levy stability criterion:

\begin{align*}
\Delta t < \frac { 1 }  { { { v_ { max } } \sqrt { \frac { 1 }  { { \Delta { x^2 } } } + \frac { 1 }  { { { { \Delta } } { y^2 } } } + \frac { 1 }  { { \Delta { z^2 } } } } } } = \Delta { t_c } \tag { 5 } 
\end{align*}

where *v*_max_ is the velocity in the medium with the smallest permittivity. We typically choose $\Delta t = 0.95 \Delta {t_c}.$ In addition, adding to the computation time, perfect match layers (Berenger, 1994) must be placed on the walls of the computational box to suppress parasitic reflections of the waves on these walls.

A preliminary numerical model of the WISDOM Vivaldi antennas was developed by Hamadi (2010); each antenna consists of two tilted wires connected to a generator, which produces a sino-gaussian signal in agreement with the characteristics of WISDOM. We added resistive loads along these wires following the works of Wu and King (1965) and Shen and King (1965) to better reproduce the actual radiation pattern of the instrument.

The radar environment is modeled by assigning electrical properties (dielectric constant, electrical conductivity) to each cell of the 3D mesh of the computation box. Magnetic properties can also be introduced, but the present work is restricted to nonmagnetic materials, which is supported by the permeability values close to the vacuum one, which are presented in Table 3. To test WISDOM's ability to respond both to the scientific requirements (characterize the subsurface, find structures of interest, and retrieve the geologic context) and the engineering constraints (localize potential hazards for the drill) of the ExoMars mission, we investigated three geological models: a buried channel, a wedge, and a subsurface that included buried blocks. To be imported in TEMSI-FD, these models were converted into geoelectrical models by using expected values of the electrical properties inferred from laboratory measurements on martian analogs (see section 5.1).

The typical model includes the antenna system (transmitting and receiving antennas), lying 30 cm above the modeled environment. To simulate WISDOM operations during the ExoMars mission, the two antennas are moved along a profile, and a numerical simulation is run every 10 cm. All radar traces (*i.e.,* EM field at the receiving antenna as a function of time) obtained are then combined into a 2D radargram. In this study, we only simulated WISDOM operations in copolar mode (*i.e.,* with parallel transmitting and receiving antennas).

#### 5.2.2. Numerical simulations of WISDOM's performance in geologically realistic environments

Buried landforms are expected in sedimentary environments. Oxia Planum, the selected landing site for ExoMars 2020, exposes a sequence of sedimentary rocks (Quantin *et al*., 2016), whose structure will differ, depending on their formation processes (fluvial, lacustrine, aeolian, volcanic, etc). In this study, we consider two types of buried landforms that may be present in the clay-rich sediments of Oxia Planum: a buried channel and a desiccation wedge.

##### 5.2.2.1. Buried channel

**FIG. 5.**
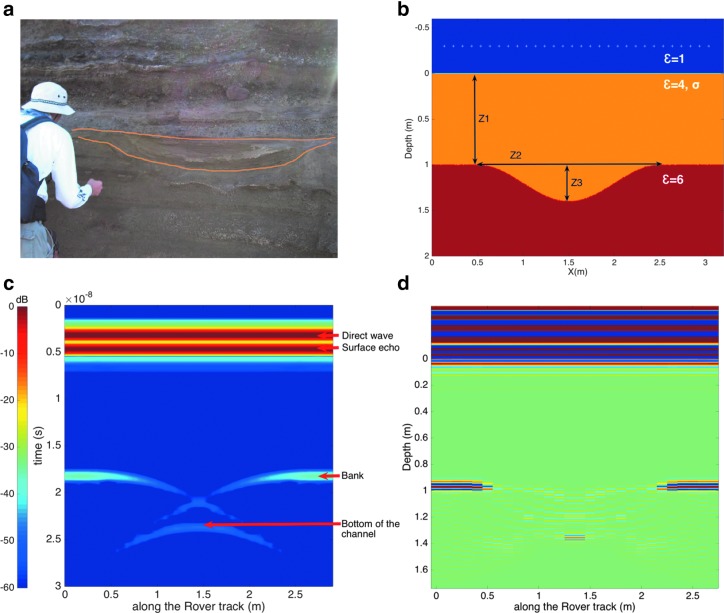
**(a)** Potential martian analogue of a buried channel in the Ka'u Desert, Hawaii, **(b)** geoelectric model of the buried channel, assuming a channel with steeply sloping sides beneath a 1-m-thick depositional layer with a dielectric constant of 4 and conductivity of 10^−2^ S/m, **(c)** WISDOM simulated radargram for the geolectrical model shown in **(b)**, **(d)** WISDOM migrated radargram.

Buried channels are often observed in the terrestrial sedimentary record as evidence of fluvial processes that eroded or deposited sediments. For instance, the layered ash deposits in the Ka'u Desert (Hawaii) are formed by intermittent aerial deposition. Between the ash deposition events, surface runoff eroded channels that were then buried by subsequent episodes of ash deposition (Fig. 5a) (Craddock *et al*., 2012). Burial is especially likely for shallow channels, whose depth is less than the thickness of the ash layer deposit (typically tens of centimeters). Such buried landforms are expected in Oxia Planum, where orbital imaging of eroded sedimentary deposits has revealed inverted channels that confirm fluvial erosional and sedimentary processes occurred at the same time (Quantin *et al*., 2016). The ability of GPR to detect buried channels on Mars has already been demonstrated by the SHARAD orbital sounder on the Mars Reconnaissance Orbiter, which revealed kilometer-scale evidence of buried channels in the Elysium volcanic region (Morgan *et al*., 2013). Small channels are indicative of surface runoff and indicate that conditions were once favorable for atmospheric precipitation. The identification of such landforms by WISDOM will provide unique insights into environmental conditions that prevailed during the time the channels were formed.

To test the ability of WISDOM to detect and characterize buried channels, we modeled the radar profile it would acquire over a small-sized buried channel, using the EM modeling software, TEMSI-FD. The simulation assumed a buried channel, eroded into a substrate with a dielectric constant ɛ′ of 6, overlain by a layer of volcanic ash with a real permittivity ɛ′ of 4, and varying conductivity and depth (Fig. 5b). The depth-to-width ratio of the channel was set to 0.22 (width: Z2 = 1.8 m, depth: Z3 = 0.4 m), which is in agreement with a potential analog discovered in the Ka'u Desert (Fig. 5a). We ran a number of simulations, varying both the conductivity (in the range of 10^−4^–10^−2^ S/m) and thickness Z1 (1, 2, 2.5 m) of the depositional layer, to determine the range of conditions (in terms of σ, Z1) over which the buried channel remained detectable by WISDOM.

The WISDOM simulated radargram obtained for Z1 = 1 m and σ = 10^−2^ S/m (Fig. 5c) clearly shows echoes from the bottom and the two banks of the channel. The echo from the bottom takes the form of a complete hyperbola, while the echoes from the channel banks create hyperbolic branches (*i.e.,* half of complete hyperbola). For the case shown in Fig. 5, the margins of the channel are too steep to be detected. WISDOM would, however, clearly detect these margins if their slope were gentler (namely up to 20°). Note that this limit would be pushed to 35° in the case of rough margins. To reconstruct the shape of the channel from the simulated data, we use Gazdag's phase-shift migration with matGPR (Tzanis, 2006, 2010) to focus the hyperbolas into punctual reflectors, resulting in Fig. 5d, from which the width and depth of the channel can be readily inferred.

For all investigated (Z1, σ) combinations, amplitudes of the echoes arising from the banks and bottom of the channel are less than 80 dB below the surface echo (*i.e.,* the instrument dynamic, see Section 3). This implies that WISDOM is able to detect a buried channel and, more generally, interfaces below 2.5 m of a moderately absorbing medium (with conductivity as high as ∼10^−2^ S/m, *e.g.,* clay-rich material).

##### 5.2.2.2. Desiccated wedge

Polygonal patterns are observed at many scales on the surface of Mars. Some of these are suspected to be desiccation polygons (El-Maarry *et al*., 2014) that form when wet clay-rich sediments deposited in paleolacustrine environments dry out, which causes the clay to shrink and crack. On Mars, clay and chloride-bearing rocks frequently show polygonal surface patterns, with widths ranging from centimeters to tens of meters. As desiccation polygons are associated with formerly wet environments, they are primary targets for *in situ* exploration. In Oxia Planum, orbital spectral imaging has revealed the presence of a clay-bearing unit with meter-size polygons (Quantin *et al*., 2016). Thus, WISDOM could potentially encounter polygon wedges, whose detection would be crucial to establishing both the nature of the paleoenvironment recorded in the local sediments and a guide for sampling with the drill.

**FIG. 6.**
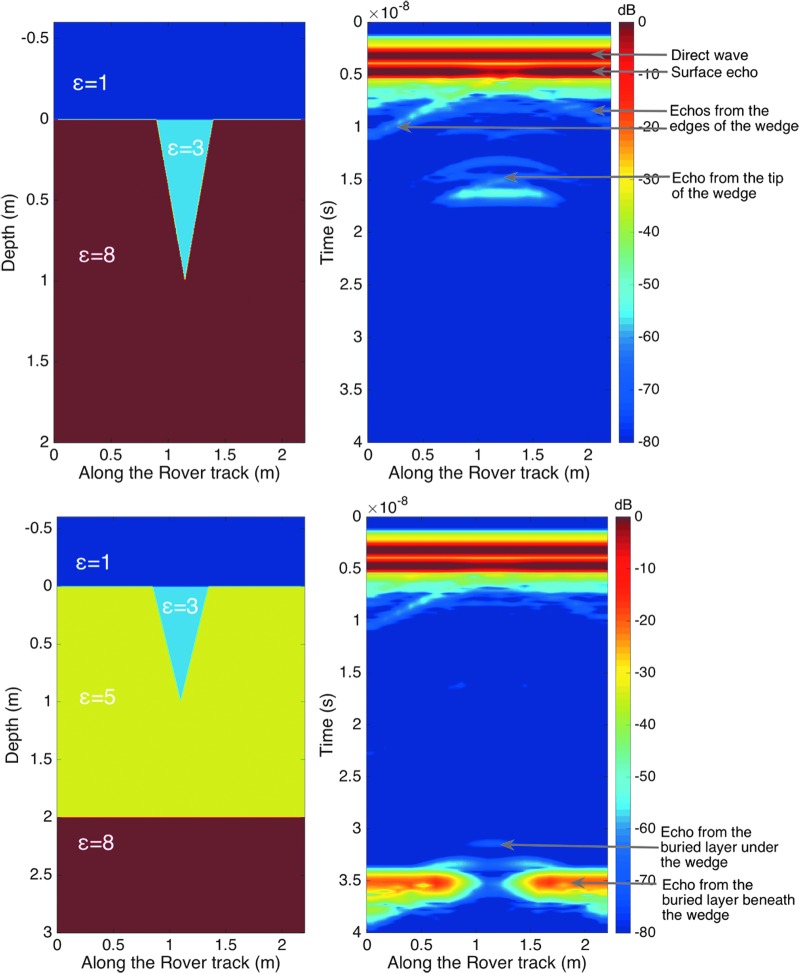
Numerical simulations of WISDOM operations in the presence of a wedge without, and with, an underlying layer (top and bottom panels, respectively). In each case, the left figure displays the geoelectric model imported under TEMSI-FD (with a mesh size of 5 mm) and the right figure shows the associated WISDOM simulated radargram. Simulation was conducted with a 10-cm sounding interval.

To test WISDOM's ability to detect and characterize these features, we simulated its operation over two models of the subsurface with a 1-m-deep and 50-cm-wide desiccation wedge (which is the characteristic boundary of a desiccation polygon). In the first case (Fig. 6, top panels), the dielectric contrast between the material filling the wedge (ɛ′ = 3) and the ground (ɛ′ = 8) is strong enough to generate a hyperbola signature whose amplitude is larger than the dynamic of the instrument. The time of arrival of the echo at the summit of this hyperbola combined with the knowledge of the wedge dielectric constant derived from the surface echo can be used to retrieve the depth to the bottom of the wedge (*i.e.,* 1 m). In the second case (Fig. 6, bottom panels), the echoes arising from the tip of the wedge are too weak to be detectable (the dielectric contrast between the material filling the wedge (ɛ′ = 3) and the ground (ɛ′ = 5) being small). However, the difference in terms of time of arrival of the echoes from an underlying layer beneath and under the wedge can still be used to infer the depth of the wedge.

##### 5.2.2.3. Buried blocks

**FIG. 7.**
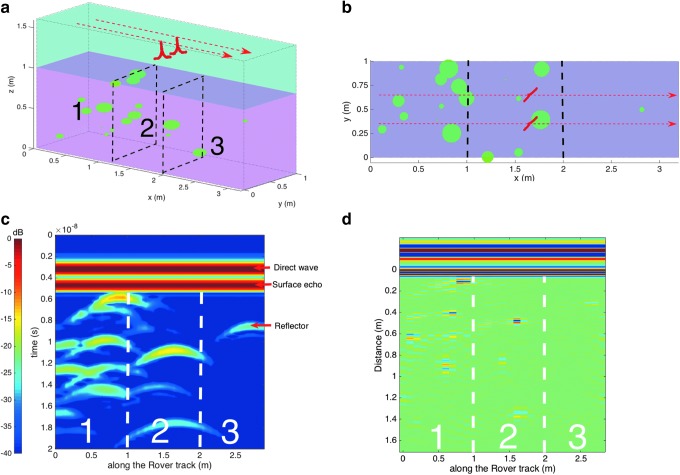
**(a)** TEMSI-FD 3D computational box showing the WISDOM antennas (in red) lying 30 cm above the ground (in pink), which is divided into three areas with different densities of rocks (in green). The red arrows represent the 3.3-m-long track of WISDOM. **(b)** View from above of the computational box. **(c)** WISDOM simulated radargram, **(d)** WISDOM migrated radargram. 3D, three-dimensional.

Once a location of high scientific interest is located, WISDOM will play a key role in identifying the most promising and safest places to drill and sample. The greatest concern is the presence of buried rocks that could damage the ExoMars drill. To illustrate WISDOM's ability to detect subsurface hazards, we simulated its operations over ground while possessing a range of embedded rock sizes and three different densities (Fig. 7).

The rocks are modeled as spheroids with a semimajor axis in the range 1–10 cm and a sphericity of 0.5. They are assumed to be made of a material with real relative permittivity ɛ′ of 6 and are embedded in a medium with ɛ′ of 4. Losses by absorption are neglected. We used a 3D computational box of 3.3 × 1 × 2.6 m with a mesh size of 5 mm in the 3 dimensions (Fig. 7a) to simulate wave propagation with TEMSI-FD. In accordance with the Rover RSM, measurements are simulated every 10 cm to build a radargram (shown in Fig. 7c). Each hyperbola is the signature of a buried reflector. A total of 16 of the 19 modeled rocks are detected (only the three 1-cm rocks are missed), resulting in the conclusion that areas 1 and 2 are to be avoided, while area 3 is relatively safe.

Gazdag's phase-shift migration process further enables us to rapidly evaluate the distance between the reflectors and the antennas (Fig. 7d). However, unless the rock is right at the vertical of WISDOM antennas, this distance can be much larger than its actual depth. The accurate location of the reflectors as well as information on their shape can be subsequently retrieved by conducting polarized measurements or by moving the sounding position of WISDOM.

### 5.3. Field tests in natural environments

A prototype that is representative of the WISDOM Flight Model (same design, mass, volume, power consumption, and function) is currently being used for field testing. Because liquid water is highly conductive and a strong reflector, its presence in soil greatly attenuates the propagation of radar waves. Therefore, we have conducted our field tests in either dry (Atacama Desert in Chile, Etna in Italy) or cold (glacier in the Alps, Dachstein ice caves in Austria) environments. This testing, in well-characterized environments, is critically important for validating the data processing and analysis tools to be used and for a better understanding of the instrument's likely performances on Mars.

**FIG. 8.**
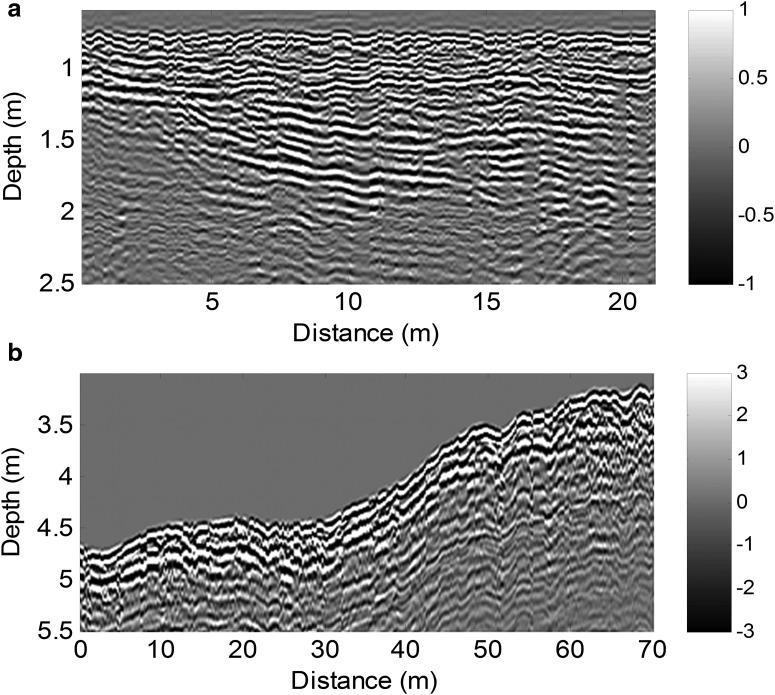
WISDOM radargrams that reveal **(a)** two superimposed events of pyroclastic layered deposits (Etna, Italy), the most recent one corresponding to a global layer of ∼50 cm horizontally stratified, and **(b)** a small ∼50-cm-deep and ∼7-m-wide dried channel bed in the Atacama Desert (Chile) at about 30 m from the left. The amplitude is in arbitrary unit.

The data collected in various environments (Fig. 8) have demonstrated WISDOM's ability to resolve shallow fine-scale structures and thus provide clues for understanding the geology and geological evolution of the site. During the SAFER experiment in Atacama, WISDOM was used to sound an area where a buried channel bed was exposed at the surface. WISDOM succeeded in detecting and following the bottom of the channel beneath ∼50 cm of sediment (Fig. 8b). Colocalization with surface data allowed for a successful 3D representation of the site. The radargrams in Fig. 8 were obtained by a simple time-to-depth conversion assuming a uniform permittivity value in the subsurface (corresponding to data product #6bis of Table 4). The permittivity value used was estimated from the surface echo, namely 5.7 for the Etna (a) and 3.7 for Atacama (b).

**FIG. 9.**
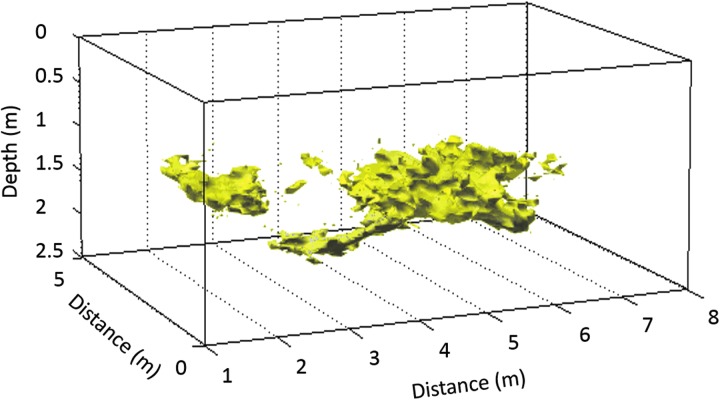
3D reconstruction of bedrock beneath a 1.5-m-thick ice layer (ice caves in Dachstein). The ice layer is not represented in the figure (modified from Dorizon *et al*., 2016).

Field testing in the ice caves of Dachstein, Austria, and volcanic ash deposits at Mt. Etna demonstrated that by acquiring data in a grid pattern, WISDOM soundings can be used to construct a 3D representation of buried interfaces, whether beneath ice or sediments. Because the radiation pattern of the antennas is directional, the soundings performed with the two copolarization configurations can be used to constrain the 3D location of buried blocks (Dorizon *et al*., 2016). An example of a 3D reconstruction of bedrock beneath a layer of ice and an example of a 3D localization of individual small rocks embedded inside the ice are presented in Fig. 9.

**FIG. 10.**
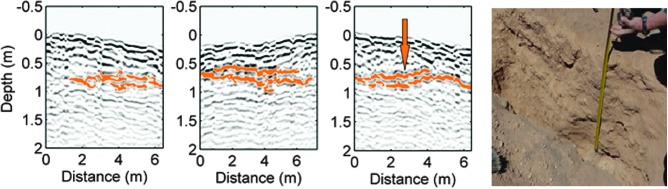
Three parallel sounding profiles obtained by WISDOM when the ExoMars Rover prototype was commanded to perform a grid during the SAFER experiment in Chile. The strong reflector, detected beneath ∼70 cm of thinly layered sediment, is highlighted in orange. The picture on the right shows the same strong sedimentary reflector exposed after digging. The orange arrow shows the location where the depth measurement was performed. SAFER, Sample Acquisition Field Experiment with a Rover.

In addition to testing WISDOM's capabilities, field campaigns are also underway to refine team, instrument, and rover operations (rover navigation, payload operations, fast data processing, and interpretation) in preparation for landing on Mars in 2020. During the SAFER experiment in 2013, a preliminary version of ESA's ExoMars Rover with onboard prototypes of three ExoMars instruments—WISDOM, PanCam, and Close-UP Imager (CLUPI)—was operated in Chile from a remote control center at the Satellite Applications Catapult facility in Harwell, United Kingdom. This experiment allowed us to operate WISDOM under operational conditions similar to those expected during the ExoMars mission with regard to data processing, decision-making, and operation planning. A grid was performed that allowed us to detect a sedimentary layer ∼70 cm below the surface, whose presence and depth were confirmed by excavation of an observation pit (Fig. 10).

## 6. Conclusion

The first GPR to be selected for a Mars Rover mission, WISDOM, will be used in combination with ExoMars Rover's PanCam imaging system and ADRON neutron spectrometer to perform large-scale scientific investigations of the landing site and rover environment. These data, supplemented by those acquired by other instruments of the Pasteur payload, will provide important information on the nature of the shallow subsurface that is essential to understanding the processes and environmental conditions responsible for its formation, as well as its past and present habitability.

WISDOM will address the principal objectives of the ExoMars mission by providing high-resolution observations of the stratigraphy and structure of the shallow subsurface that will be crucial to the identification of optimal drilling sites, where organic molecules—and other potential evidence of life—are most likely to be found. WISDOM will also help to ensure the safety of drilling operations by identifying potential hazards and successful retrieval of subsurface samples.

To ensure accurate interpretation of WISDOM data to be returned from Mars, field tests are currently being conducted with a fully functional instrument prototype in a wide variety of arid and cold climate environments. The resulting database of well-characterized terrestrial analogue environments will improve our ability to characterize similar environments on Mars and provide valuable experience in procedures and data analysis for the team. Ongoing field tests with other instruments of the Pasteur payload (including PanCam, CLUPI, ADRON, and Ma_MISS) will continue to be conducted to enhance their operational and scientific synergies.

## Abbreviations Used

ADC: analog-to-digital converter
CLUPI: Close-Up Imager
EC: experiment cycle
EM: electromagnetic
EU: electronics unit
GPR: ground-penetrating radar
iFoV: instantaneous field of view
IFT: inverse Fourier transform
Ma_MISS: Mars Multispectral Imager for Subsurface Studies
PanCam: panoramic camera
RSL: recurrent slope lineae
RSM: reference surface mission
SAFER: Sample Acquisition Field Experiment with a Rover
SF: step frequency
SNR: signal-to-noise ratio
WISDOM: Water Ice Subsurface Deposit Observation on Mars
